# Case Report: Reflections on “Tumor Plop” in a febrile hemodialysis patient who had atypical presentation of atrial myxoma as infective endocarditis

**DOI:** 10.3389/fcvm.2025.1645356

**Published:** 2025-09-29

**Authors:** Fangzhong Huang, Jian Huang, Xingzhen Zhang, Shuangqing Li, Lingli Zhu, Jun Ying, Fang Zhou, Yingxin Zhang, Xuchun Xu

**Affiliations:** Department of Nephrology, Jinhua Municipal Central Hospital (Affiliated Jinhua Hospital, School of Medicine, Zhejiang University), Jinhua, Zhejiang, China

**Keywords:** infective endocarditis, hemodialysis, tumor plop, atrial myxoma, embolic stroke

## Abstract

We report a rare case involving a 43-year-old male on long-term hemodialysis who developed infective endocarditis (IE) accompanied by a diastolic “tumor plop” sound and a large atrial mass, a presentation more commonly linked to atrial myxomas. The patient initially experienced an upper respiratory tract infection caused by Type I parainfluenza virus, which progressed to severe pneumonia. During hospitalization, physical examination revealed an atypical diastolic “tumor plop” sound, prompting further evaluation. Echocardiography identified a sizable atrial mass measuring 51 mm × 33 mm × 32 mm, which oscillated between the left atrium and ventricle throughout the cardiac cycle. Blood cultures confirmed a bloodstream infection with *Rothia dentocariosa*. Concurrently, the patient suffered an embolic stroke, likely due to detachment of the cardiac mass. Clinical findings supported a diagnosis of IE with embolic stroke caused by the atrial mass, rather than an atrial myxoma. The patient underwent surgical removal of the mass along with a full course of antibiotic therapy, which led to a significant improvement in clinical status. This case demonstrates that the clinical features of IE in patients receiving hemodialysis may resemble those of an atrial myxoma, including the unusual “tumor plop” sound. It also illustrates the diagnostic and therapeutic challenges encountered in such cases, where rapid identification and treatment are essential for improving outcomes.

## 1 Introduction

Infective endocarditis (IE) is a life-threatening condition, particularly in individuals with comorbidities such as end-stage renal disease undergoing hemodialysis. These patients face a higher risk of IE due to repeated vascular access, prolonged catheter placement, and frequent exposure to diverse pathogens (1–4). IE commonly presents with fever, the emergence of a new heart murmur, and a risk of embolic events, necessitating a strong clinical suspicion for timely diagnosis (5, 6). A defining feature of IE is the development of vegetations on cardiac valves. However, these vegetations can sometimes mimic other intracardiac masses, such as atrial myxomas, thrombi, and lesions associated with rheumatic heart disease, both clinically and radiographically (see Table 1).

**Table 1:** Summary of potential differential diagnoses for atrial mass.

Year	Author	Age/sex	Clinical features	Complications	Pathogen	Main Treatment
2014 (11)	Anshuan Darbari	6/male	High fever, erythematous rashes, dyspnea, hepatomegaly, tumor plop	Pulmonary hypertension, vegetation on tricuspid leaflets	*Staphylococcus aureus*	Surgical excision of vegetation, valve repair, 6-week antibiotic therapy (ceftriaxone/cefixime)
2016 (20)	Talita G. Salani	19/female	Asymptomatic, right atrium mass	None	None (thrombus)	Anticoagulation therapy, no surgical intervention
2017 (14)	Jun Xu	63/male	Progressive dyspnea, leg edema, fever, tumor-like mass in the left atrium	Heart failure, bacterial vegetation, severe aortic insufficiency	*Corynebacterium striatum*, *Acinetobacter baumannii* (sputum)	Double valve replacement, daptomycin, linezolid
2018 (7)	Gerald paul Fitzgerald.	23/male	Fever, weight loss, night sweats, fatigue, general malaise, tumor plop	Necrotic and infected atrial myxoma mimicking IE	*Streptococcus viridans*	Empirical antibiotics; surgical excision of atrial myxoma
2021 (8)	Ovidiu Stiru	63/male	Chest pain, dyspnea, cardiac mass adjacent to dialysis catheter tip	Right atrial thrombosis, misdiagnosed as myxoma	None	Surgical excision of mass (histopathology confirmed thrombus)
2023 (21)	Mohsen Gholinataj Jelodar	40/male	Fever, mental confusion	Septic pulmonary embolism, vegetative infected thrombus	*Staphylococcus aureus*, COVID-19	Vancomycin and gentamicin, surgical resection of the right atrial mass (histopathology confirmed myxoma)
2024 (13)	Ying-Chi Shen	55/male	Acute vertigo, unsteady gait, cyanotic fingers, Janeway lesions, Osler nodes, splinter hemorrhages	Posterior circulation embolic stroke, multiple infarctions	None (mimicry of IE by atrial myxoma)	Early surgical excision of left atrial myxoma; dual antiplatelet therapy
2025 (10)	Bhavik Sandip Shah	Teenage/female	Dyspnea, palpitations, a rumbling mid-diastolic murmur	Heart failure	None (RHD, IE, co-existing atrial myxoma)	Mitral valve replacement and complete excision of the atrial myxoma

IE, infective endocarditis; RHD, rheumatic heart disease.

On rare occasions, IE can result in the formation of a cardiac mass that closely resembles an atrial myxoma (7–10). Atrial myxomas, though benign, are often identified by the distinctive diastolic “tumor plop” sound during auscultation (11, 12). When IE-associated vegetations become sufficiently large and mobile, they may produce a similar murmur, potentially leading to the mistaken diagnosis of a myxoma. This overlap complicates diagnosis, especially when both clinical presentation and auscultation findings align with those typically seen in atrial myxomas.

In the present case, the patient exhibited a large mass in the left atrium, a “tumor plop” sound, and an embolic stroke caused by mass detachment, raising initial concerns for atrial myxoma. However, detailed evaluation through echocardiography and microbiological testing, along with a review of the patient's medical history, revealed the mass to be infective vegetation rather than a myxoma. Although instances of IE mimicking atrial myxoma have been documented (7, 10, 13, 14), such presentations remain uncommon among hemodialysis patients. This case may contribute valuable clinical perspective for diagnosing and managing similar presentations in this high-risk group.

## 2 Case presentation

### 2.1 Medical history

A 43-year-old male with a history of more than 3 years of regular hemodialysis was admitted to the nephrology department with a 2-week history of fever on May 13, 2024. Informed consent was obtained, and the study received approval from the hospital's ethics committee. The patient had been diagnosed with stage 5 chronic kidney disease over 3 years prior and had undergone placement of a semi-permanent deep venous catheter for long-term hemodialysis on July 31, 2020. He subsequently received scheduled dialysis. On December 28, 2020, an arteriovenous fistula was surgically created. The hemodialysis catheter was removed on May 17, 2021, and dialysis was continued using the arteriovenous fistula, with treatments performed regularly on Tuesdays, Thursdays, and Saturdays.

The patient began experiencing fever with chills on 2024 May 1, 2 weeks prior to admission, with a maximum temperature of 38°C. He reported a non-productive cough but denied chest tightness, dyspnea, nausea, vomiting, lower limb edema, diarrhea, abdominal pain, rash, or joint pain. He first visited the fever clinic at our hospital, where “epidemic acute upper respiratory viral infection” was suspected. At that time, heart and lung auscultation findings were normal. He was prescribed cephalosporin antibiotics and diclofenac sodium suppositories. However, after a week of ineffective treatment, his symptoms worsened, including increased chest discomfort, dyspnea, and recurrent fever. On May 13, 2024, he visited the emergency department for further care. On the same day, nucleic acid testing returned positive for Type I parainfluenza virus. Complete blood count revealed a white blood cell count of 16.29 × 10^9^/L, with neutrophils at 84.9%, lymphocytes reduced to 5.3%, eosinophils at 0.1%, and an elevated neutrophil count of 13.82 × 10^9^/L. Red blood cell count was low at 3.36 × 10^12^/L, hemoglobin was reduced to 98 g/L, and platelets measured 114 × 10^9^/L. Despite receiving ertapenem 1 g intravenously once daily and oseltamivir 75 mg orally once daily for 1 week, the patient's fever and chest discomfort persisted. He was therefore admitted for further investigation under the working diagnosis of “fever of unknown origin”.

**Figure 1:**
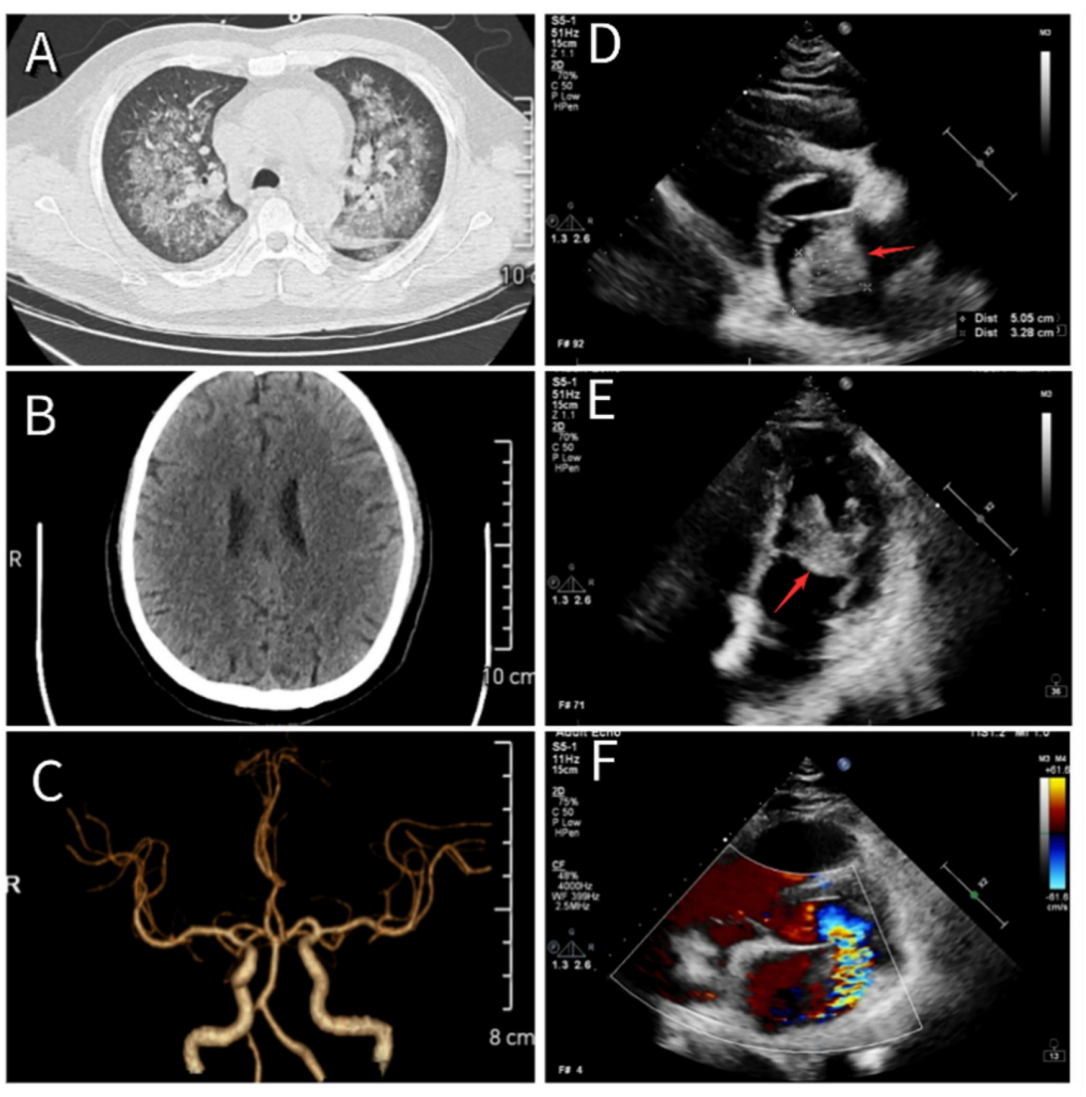
**(A)** Chest CT showing changes consistent with severe pneumonia. **(B)** Cranial CT showing no evidence of cerebral infarction. **(C)** Cranial CTA revealing normal cerebral vasculature. **(D,E)** Echocardiographic images showing a newly identified atrial mass located at the root of the anterior mitral leaflet, measuring approximately 51 mm × 33 mm × 32 mm (red arrow). **(F)** The mass moves between the left atrium and ventricle with each cardiac cycle, resulting in severe mitral regurgitation.

His medical history included more than 2 years of hypertension, managed with sacubitril/valsartan, and no other notable conditions. On physical examination, the pulse was 136 beats per minute, respiratory rate 22 breaths per minute, blood pressure 106/59 mmHg, and body temperature 38.5°C. Auscultation revealed widespread fine crackles in both lungs, a regular heart rhythm, and a newly detected low-pitched diastolic murmur at the apex. No significant edema was present in the lower limbs. A continuous blowing murmur was heard over the arteriovenous fistula in the left forearm. The initial diagnoses were infectious fever, severe pneumonia (Figure 1A), chronic kidney disease stage 5, secondary hypertension, and hemodialysis status.

### 2.2 Diagnosis and treatment course

Based on the diagnosis of infectious fever and severe pneumonia, the patient was started on intravenous piperacillin-tazobactam (4.5 g every 12 h) and vancomycin (0.5 g once daily) to provide broad coverage for both Gram-positive and Gram-negative organisms, along with supportive therapy. On the evening of May 24, 2024, he developed acute confusion, delayed responses, and significant chest tightness accompanied by dyspnea. Emergency consultations with neurology and cardiology were initiated. Given the brief onset time, cranial computed tomography angiography (CTA) showed no apparent vascular abnormalities or cerebral embolic lesions (Figures 1B,C). Cardiac auscultation by the cardiologist revealed a characteristic “tumor plop” sound at the apex, leading to immediate bedside transthoracic echocardiography. The scan detected a heterogeneous echogenic mass in the left atrium, approximately 51 mm × 33 mm × 32 mm in size (Figures 1D,E), raising suspicion for either a vegetation associated with IE or an atrial myxoma. Blood cultures collected on May 25 confirmed a bloodstream infection with *Rothia dentocariosa*. Serial tests showed a rising trend in cardiac troponin, persistent fever, elevated inflammatory markers, and other abnormalities (see Table 2).

**Table 2:** Summary of key clinical indicators during hospitalization.

Indicators	5–13	5–23	5–24	5–25	5–26
Temp (°C)	38	38.5	38.2	38.1	38
BP (mmHg)	123/70	106/59	113/67	118/71	107/62
Alb (g/L)	34.1	24	25.1	31.9	28.5
Hb (g/L)	98	94	84	86	72
hs-CRP (mg/L)	126	106	77	80	119
PCT (ng/ml)	7.29	7.01	4.64	4.70	4.52
TnI (μg/L)	null	0.145	0.085	0.085	2.250
NT-pro BNP (g/ml)	null	1,488	907	879	500

5–13 refers to May 13, 2024; subsequent dates follow the same format. Temp, temperature; BP, blood pressure; Alb, albumin; Hb, hemoglobin; hs-CRP, high-sensitivity C-reactive protein; PCT, procalcitonin; TnI, troponin I; NT-pro BNP, N-terminal pro B-type natriuretic peptide.

In light of the patient's worsening condition, a multidisciplinary consultation was held on May 25. Specialists from cardiology, cardiothoracic surgery, infectious diseases, echocardiography, intensive care, and respiratory medicine departments participated. After review, the clinical team reached a consensus diagnosis of IE complicated by embolic stroke due to detachment of the atrial mass. Cardiac valve surgery was advised once the infection became controlled. Following discussions with the patient and his family, transfer arrangements were made. On May 26, he was discharged and referred to the First Affiliated Hospital of Zhejiang University for further treatment.

### 2.3 Follow-up summaries

**Figure 2:**
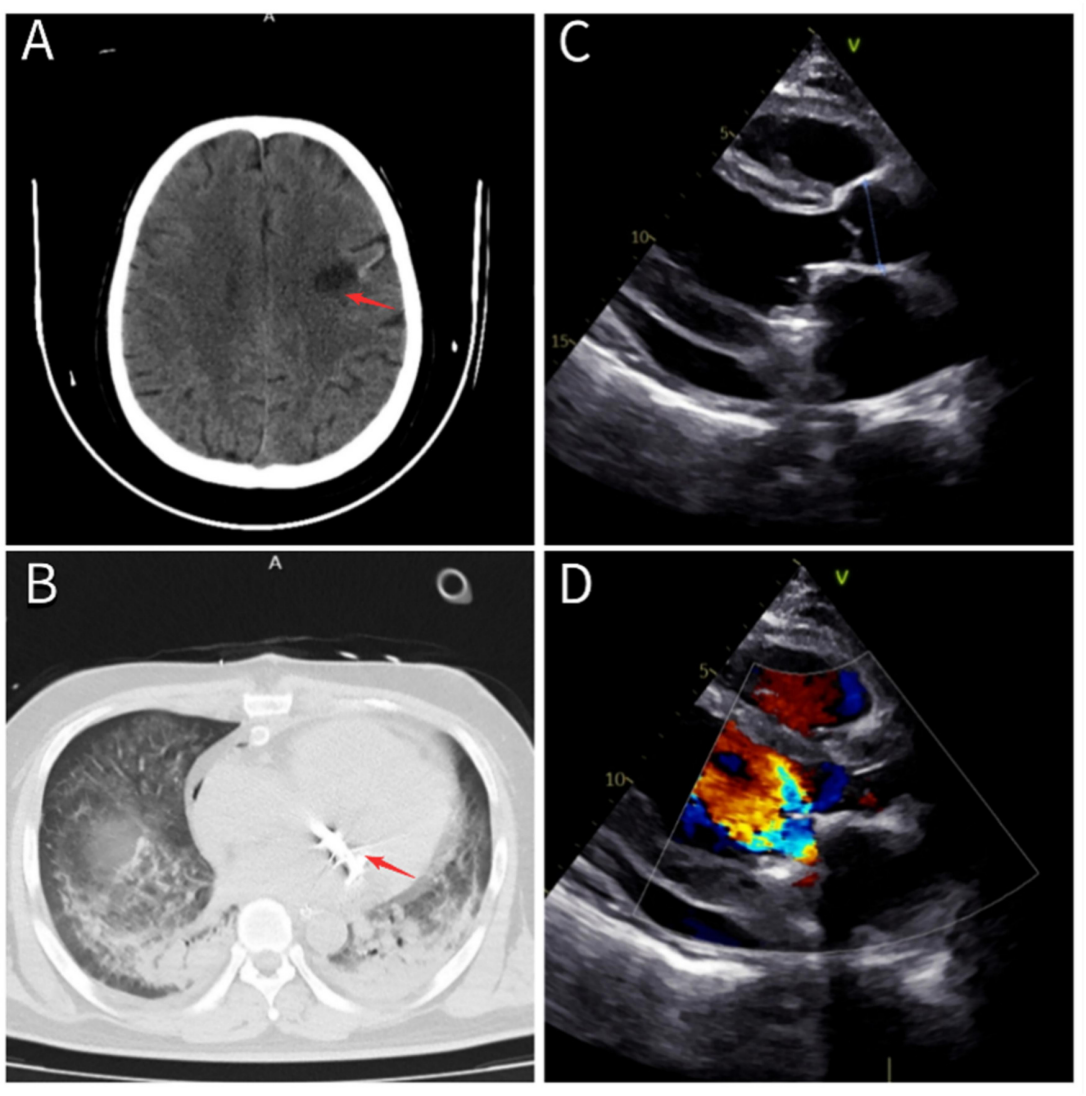
**(A)** Cranial CT revealing an infarction in the left frontal lobe (red arrow). **(B)** Chest CT confirming correct placement of the mitral mechanical valve (red arrow). **(C,D)** Echocardiographic images demonstrating normal function of the mitral mechanical valve with no residual intracardiac masses.

After transfer to the First Affiliated Hospital of Zhejiang University, the patient's condition worsened further. He developed increasing chest tightness and dyspnea, accompanied by recurrent confusion and delayed cognitive response. Repeated cranial computed tomography (CT) scans during hospitalization revealed a low-density lesion in the left frontal lobe (Figure 2A), consistent with cerebral infarction. Due to the significant risk of further cerebral embolization from detachment of the large atrial mass and ongoing damage to the mitral valve, cardiac valve surgery was deemed absolutely necessary (6). Taking into account the patient's young age and moderate financial situation, mitral valve mechanical replacement, offering longer durability, was chosen to avoid repeated surgeries. The procedure was performed on June 6, 2024. A postoperative chest CT conducted on June 7 confirmed the prosthetic valve was correctly positioned (Figure 2B). The patient was treated postoperatively with intravenous piperacillin-sulbactam (4.5 g once daily) and daptomycin (0.5 g once daily).

After being discharged on 2024 June 19, the patient resumed maintenance hemodialysis at our hospital's dialysis center. Given the patient's severe pneumonia and sputum culture positive for Klebsiella pneumoniae, the same antimicrobial regimen, piperacillin-sulbactam 4.5 g IV daily and daptomycin 0.5 g IV daily, was continued to complete a 4-week course. On follow-up transthoracic echocardiography performed on July 9, the mitral prosthetic valve demonstrated normal function, with no residual intracardiac masses (Figures 2C,D).

## 3 Discussion

Infective endocarditis (IE) remains a serious and life-threatening condition in patients with end-stage renal disease undergoing maintenance hemodialysis (1). This group faces increased vulnerability due to factors such as prolonged catheter use, repeated vascular access procedures, and weakened immune responses. The incidence of IE is increased in dialysis patients, yet its timely identification is often hindered by nonspecific symptoms like fever, fatigue, and new-onset heart murmurs (15). The clinical picture is often clouded further by common comorbidities in this population, including anemia, malnutrition, and calcium-phosphorus imbalance (14). In the present case, the intracardiac mass appeared similar to an atrial myxoma on echocardiography and was accompanied by a “tumor plop” sound, blurring the distinction between infective vegetation and a primary cardiac tumor.

The “tumor plop” is a rare auscultatory feature in IE (7, 11), more commonly associated with a large left atrial myxoma. In contrast, cardiac tumors' plops are typically persistent, not acute-onset, and unassociated with fever or elevated inflammatory markers. Additionally, definitive diagnosis of cardiac tumors requires histopathology. In this case, the new-onset “tumor plop” detected between May 1 and 24, combined with the patient's recurrent fever during this period and long-standing hemodialysis history, heightened the consulting cardiologist's suspicion of IE. This clinical constellation prompted a strong recommendation for immediate bedside cardiac ultrasound, with particular attention to the source of the “tumor plop”. The ultrasound revealed a large left atrial mass, providing a critical diagnostic clue for confirming IE. While the excised left atrial mass was not subjected to histological examination, this represents a limitation of this case report. However, based on the patient's clinical background, history, and diagnostic results, IE was considered the more likely cause of the mass.

Several factors likely contributed to the development of the large atrial mass in this patient. Long-term hemodialysis and repeated vascular access procedures, such as arteriovenous fistula punctures, increased the risk of bloodstream infections leading to IE (16). In addition, the previous extended use of a semi-permanent catheter, maintained due to the patient's reluctance to undergo fistula punctures because of pain, further increased susceptibility to infection (17). Compounding this, recurrent febrile episodes following an acute upper respiratory tract infection were not promptly or adequately managed with appropriate antibiotics. The delay in treatment and diagnosis likely played a major role in the progression of IE and the formation of the atrial mass (18).

Our clinical experience with this case provides several key observations. First, regardless of the specific patient population or pathogen, when vegetation in IE becomes large enough and moves between the atrium and ventricle with positional changes, it can produce a “tumor plop” sound similar to that of an atrial myxoma. Second, while the causes of an atrial mass may include IE, thrombus formation, primary cardiac tumors (such as atrial myxoma), or even a combination of these (see Table 1), a differential diagnosis is still possible by assessing infection markers like blood cultures, echocardiographic features, detailed medical history, and physical examination. Third, the presence of a “tumor plop” sound in patients with IE on hemodialysis may suggest a delay in diagnosis and treatment. This is because a mass of sufficient size to produce such a sound typically forms only after prolonged infection.

As such, persistent intermittent fever in hemodialysis patients should not be overlooked or simply attributed to acute upper respiratory tract infection. In many cases, the respiratory infection may act as a trigger or initial clinical indicator rather than the underlying cause. Early and comprehensive clinical evaluation (19), including careful history-taking, physical examination, and echocardiography, might have led to earlier detection of the cardiac mass and potentially prevented the progression to IE and its complications. When severe complications arise in IE among hemodialysis patients, such as heart failure or cerebral embolism as seen in this case, coordinated management involving multiple specialties, timely and targeted antibiotic treatment, and appropriately scheduled surgical intervention are critical for effective care.

## 4 Conclusion

Regardless of the patient group or pathogen involved, when vegetation in infective endocarditis grows large enough and oscillates between the atrium and ventricle, it can produce a “tumor plop” sound that mimics that of an atrial myxoma. At this stage, infective endocarditis and atrial myxoma can still be distinguished using infection markers such as blood cultures, echocardiographic findings, a thorough medical history, physical examination, and histopathology. In this case, earlier diagnosis and prompt intervention might have prevented the development of the “tumor plop” sound and the resulting complications, including embolic stroke and heart failure. When serious complications do occur in hemodialysis patients with infective endocarditis, multidisciplinary management, timely and effective antibiotic treatment, and well-timed surgical intervention are essential to reducing further embolic events and improving overall prognosis.

## Data Availability

The raw data supporting the conclusions of this article will be made available by the authors, without undue reservation.
